# Ciprofloxacin Inhibits Angiotensin I‑Converting Enzyme (ACE) Activity by Binding at the Exosite, Distal to the Catalytic Pocket

**DOI:** 10.1021/acsbiomedchemau.5c00089

**Published:** 2025-06-10

**Authors:** Kyle S. Gregory, Vinasha Ramasamy, Edward D. Sturrock, K. Ravi Acharya

**Affiliations:** † Department of Life Sciences, 1555 University of Bath, Claverton Down, Bath BA2 7AY, U.K.; ‡ Department of Integrative Biomedical Sciences, Institute of Infectious Disease and Molecular Medicine, University of Cape Town, Observatory, Cape Town 7925, Republic of South Africa

**Keywords:** angiotensin I-converting enzyme, zinc metalloprotease, X-ray crystallography, inhibitor binding, enzyme structure, domain selectivity

## Abstract

Human somatic angiotensin I-converting enzyme is a key zinc metallopeptidase in cardiovascular regulation that hydrolyzes angiotensin peptides (Ang I, Ang II), as well as other vasoactive peptides, including kinins (e.g., bradykinin), substance P, the acetylated tetrapeptide Ac-Ser-Asp-Lys-Pro, and the amyloid ß-peptide. Because of its enzymatic promiscuity, ACE and its substrates and products affect many physiological processes, including blood pressure control, hemopoiesis, reproduction, renal development/function, fibrosis, and immune response. ACE inhibitors are among the most important therapeutic agents available today for the treatment of hypertension, heart failure, coronary artery disease, renal insufficiency, and general atherosclerosis. However, they need much improvement because of the side effects seen in patients with long-term treatment due to nonselective inhibition of the N- and C-domains of ACE (referred to as nACE and cACE, respectively). Here, we report that ACE activity can be inhibited by ciprofloxacin, a potent fluoroquinolone antibiotic (IC_50_ 202.7/*K*_i_ 33.8 μM for cACE). In addition, the high-resolution crystal structure of cACE in complex with ciprofloxacin reveals that it binds at an exosite away from the active site pocket, overlapping the position of a potential allosteric site with a different binding mode. The detailed structural information reported here will provide a useful scaffold for the design of future potent allosteric inhibitors.

## Graphical abstract

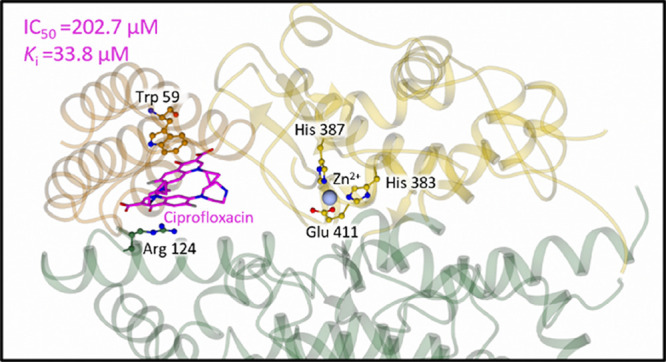

## Introduction

Angiotensin I-converting enzyme (ACE, EC 3.4.15.1) is a membrane-bound, zinc- and chloride-dependent peptidyl dipeptidase that catalyzes the conversion of the inactive decapeptide angiotensin I (Ang I) to the vasoconstrictor octapeptide angiotensin II (Ang II) by removing a carboxy-terminal dipeptide.^1^ The therapeutic success of current traditional ACE inhibitors (ACEi) for the treatment of hypertension and other cardiovascular complications lies in their ability to modulate the renin-angiotensin-aldosterone system, with pivotal roles in maintaining blood pressure and fluid balance (for reviews, see^2–5^ ). However, approximately 20–25% of patients encounter challenges in tolerating long-term treatment with traditional ACEi due to various undesired side effects. It is well documented that existing ACEi have associated side effects such as angioedema and persistent cough owing to nonselective inhibition of both domains, prompting the search for domain-specific inhibitors to mitigate adverse effects. Hence, new-generation ACEi are urgently needed with no side effects.

In somatic tissues, ACE exists as a glycoprotein known as sACE, comprising a large polypeptide chain of 1277 amino acids corresponding to the extracellular domain of the full-length membrane protein. It consists of two homologous catalytically active zinc binding centers on each N- and C-domain, referred to as nACE and cACE, respectively, with significant differences in their physiological functions. They differ in thermal stability, post-translational modifications (*N*-linked glycosylation), affinity for ACEi binding, catalytic efficiency for certain substrates, and optimal salt concentration for catalysis.^6–11^ For instance, cACE serves as the primary site for generation of the potent vasoconstrictor Ang II, whereas nACE preferentially catalyzes the inactivation of the antifibrotic peptide Ac-SDKP.

The functional characterization of ACE has been significantly advanced through the availability of high-resolution crystal structures of cACE and nACE in complex with conventional ACEi.^12,13^ These structural analyses uncovered the presence of a deeply embedded catalytic site housing a zinc ion coordinated by the conserved HEXXH zinc binding motif. This motif includes two histidine residues that coordinate the zinc ion, along with a conserved glutamate residue. Furthermore, the discovery of functionally important chloride ion binding sites in both cACE and nACE has further enhanced our understanding of their enzymatic activities. This knowledge is crucial for the design of second-generation domain-specific ACEi that can be tailored to interact with specific binding sites or active domains of ACE more efficiently, resulting in enhanced efficacy and potency without side effects.

Despite the existing knowledge, the precise mechanisms at a molecular level underpinning ACE’s substrate hydrolysis and inhibition are still not well understood. Present experimental evidence suggests the occurrence of “cooperativity” between the two domains of ACE, which may have significant effects on the pharmacological profile of domain-selective ACEi. Until recently, the only structural information available for ACE was high-resolution crystal structures of the individual domains in the “closed” conformation in the presence of ligands. These structures did not provide information regarding the “open” active site conformation prior to ligand binding. However, the recently reported crystal structure of nACE “open” in the absence of a bound ligand elucidated the motion and flexibility associated with domain opening. A comparison of the “open” and “closed” structures revealed that the two α-helices that “cap” the active site, referred to as the lid-like region of subdomain 1, pivot away from (open) and toward (closed) subdomain 2.^14^ Furthermore, the successful determination of the structures of individual domains of ACE and the full-length sACE by cryo-electron microscopy most recently reinforced that ACE is a highly dynamic molecule and undergoes intra- and interdomain conformational changes through bending, pivoting, and swinging of the interdomain linker.^15^ The cryo-EM structures showed that both domains are in an “open” conformation, extending the groove (exosite) beyond the vicinity of the active site. This would potentially support exploration of the design of allosteric inhibitors with fewer side effects than new competitive inhibitors that bind distal to the active site, thus independent of zinc chelation with increased selectivity. Furthermore, since nACE and cACE have 90% active site similarity, targeting of distal sites with less homology could allow for the development of domain-selective ACEi.

Ciprofloxacin (Cip), a fluoroquinolone antibiotic, primarily targets bacterial DNA gyrase and topoisomerase IV, resulting in the inhibition of bacterial DNA replication and repair. However, recent studies have indicated that Cip may possess additional pharmacological activities unrelated to its antimicrobial properties. One such potential activity is the inhibition of ACE as reported by Bhatti et al.,^16^ expanding the scope of this widely used antibiotic. Based on the chemical structure of Cip (Scheme 1), it was thought that it may interact with ACE domains at sites distinct from the active site (e.g., at an exosite) and the interaction could lead to the modulation of ACE activity.

**Scheme 1:**
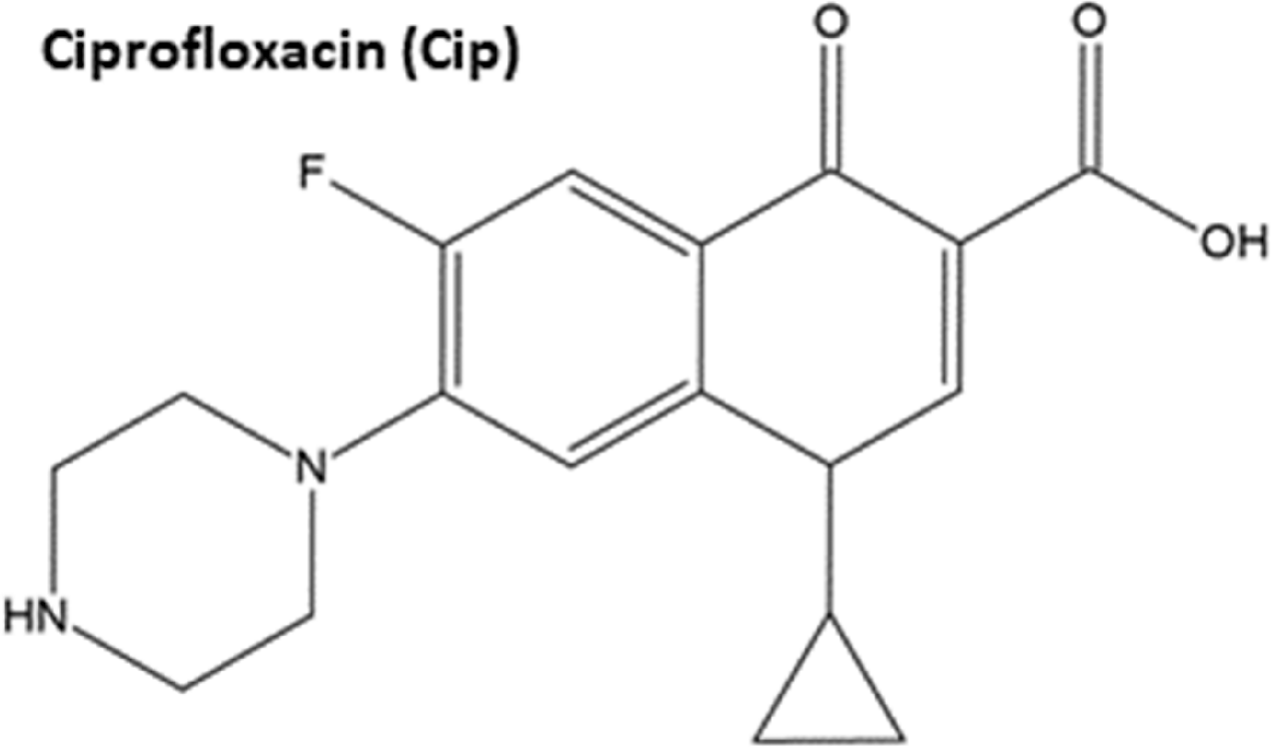
Chemical Structure of Cip

These clues prompted us to investigate whether Cip could also target individual ACE domains. Here, we demonstrate the ability of Cip to bind to and inhibit cACE. We also report the high-resolution crystal structure of the Cip–cACE complex, which could serve as the basis for future attempts to develop more potent cACE-specific inhibitory drugs.

## Materials and Methods

### Reagents

General laboratory reagents were sourced from Merck (Gillingham, Dorset, UK) or Fisher Scientific (Loughborough, Leicestershire, UK) and utilized as supplied. Reagents for molecular biology were obtained from New England BioLabs (Hitchen, Hertfordshire, UK), Stratagene, Promega (Southampton, Hampshire, UK), Novagen (Madison, WI, USA), and Sigma-Aldrich (Merck, Germany). Z-Phenylalanylhistidylleucine (Z-FHL) was procured from Bachem Ltd. (Switzerland). All reagents were sourced from UK suppliers or agents.

### Protein Production

Proteins used in the crystallographic and kinetic studies were expressed as soluble forms in mammalian CHO cells. Constructs used included the N-domain (N389, nACE) and C-domain (g1,3, cACE Ser-1 to Pro-633) of human ACE. Both proteins were purified as previously described.^6,7^

### cACE and nACE Activity Assay

The remaining activity of the enzyme cleavage of Z-FHL was measured to determine the inhibition constant of Cip. Six concentrations of Cip were prepared. The inhibitor was preincubated with an equal volume of each ACE domain (3 nM cACE and 10 nM nACE in a final reaction mixture) at 37 °C with shaking for 120 min. Thereafter, 1 mM Z-FHL in phosphate buffer (100 mM potassium phosphate buffer at pH 8.3, supplemented with 300 mM NaCl and 10 μM ZnSO_4_) was added. The hydrolysis of Z-FHL was measured as previously described.^17^ Fluorescence was measured at λ_ex_ = 365 nm and λ_em_ = 480 nm using a fluorescence spectrophotometer (Cary Eclipse, Varian).

IC_50_ values were obtained from dose-response curves plotted in GraphPad Prism v.10. *K*_i_ values were subsequently calculated by applying the Cheng–Prusoff equation (where [*S*] is the final substrate concentration of 0.5 mM Z-FHL and *K*_m_ is the concentration of the substrate (in the absence of an inhibitor) at which the velocity of the reaction is half-maximal).^18^

$$K_{i}=\frac{{\mathrm{IC}}_{50}}{1+\frac{\left[S\right]}{K_{m}}}$$

### Crystallization and Structure Determination

cACE was concentrated to 5 mg/mL and mixed with equal volumes of 20 mM Cip dissolved in water. The complex was left to equilibrate at room temperature for ∼1 h prior to setting up crystallization. Cip-cACE crystals formed by hanging-drop vapor diffusion at 16 °C in a 1 μL:1 μL with 0.1 M MIB pH 4.0, 5% glycerol, and 15% PEG 3350. Crystals were mounted onto a cryoloop and flash frozen in liquid nitrogen for X-ray diffraction data collection at 100 K. A total of 3600 images were taken at 0.1° of oscillation with an exposure time of 0.002s per image. Raw images were indexed and integrated using DIALS,^19^ with subsequent data processing performed using the CCP4 suite,^20^ including data reduction with AIMLESS, phase estimation with Phaser^21^ (using 6F9T^22^ as the model for molecular replacement), and refinement with REFMAC5 ^23^ and Coot.^24^ The Cip molecule, zinc ions, chloride ions, and purification/crystallization buffer reagents were added based on the dFo-mFc Fourier difference map. The structures were validated using Molprobity,^25^ and figures were created with CCP4MG.^26^

## Results

### Cip Inhibits cACE Activity

Bhatti et al. (2021) have explored the inhibitory effects of Cip on ACE. However, their kinetic analysis was conducted using commercially available full-length ACE, which does not differentiate between nACE and cACE. This limitation prevents a clear understanding of whether Cip exhibits selective inhibition of either domain, which is crucial given the distinct physiological roles of nACE and cACE in peptide metabolism and blood pressure regulation. To address this gap, our study investigates the domain-specific inhibition of ACE by Cip using isolated catalytic domains to determine the individual inhibition kinetics for nACE and cACE.

Cip exhibited weak inhibition of ACE, with an IC_50_ value in the micromolar range. Notably, inhibition was observed in cACE, with an IC_50_ of 202.7 μM and a *K*_i_ of 33.8 μM. In contrast, nACE displayed poor inhibition, even at high concentrations of Cip (500 μM); therefore, a reliable IC_50_ value could not be determined (Figure 1). This suggests a strong preference for cACE binding, indicating that Cip selectively interacts with cACE.

**Figure 1:**
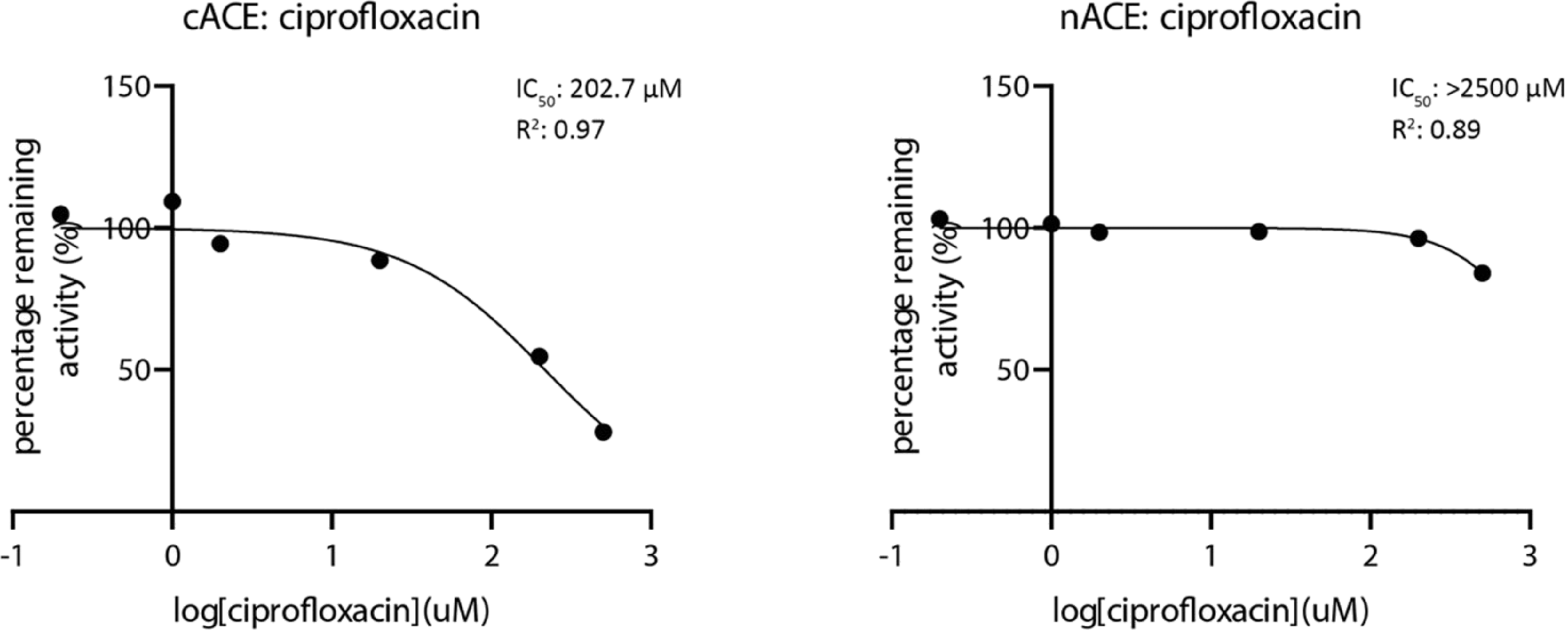
IC_50_ inhibition values and representative curves of Cip for cACE and nACE. Inhibition assays were conducted using peptide Z-FHL in the presence of varying concentrations of Cip. Cip showed a micromolar IC_50_ of 202.7 (±42) μM for cACE, but an accurate IC_50_ could not be obtained for nACE.

### Cip Binds cACE at the Exosite of the Enzymatic Pocket

To obtain structural evidence of Cip binding to ACE domains, we performed crystallization studies with both cACE and nACE. However, only crystals of cACE in complex with Cip were able to be grown, and the 3D structure was determined (crystals of nACE were produced, but they lacked electron density indicative of Cip binding). The data were collected at the Diamond Light Source (Didcot, UK), and the structure was determined by molecular replacement with our previously reported cACE structure as a search model (PDB code 6F9T).^22^ The Cip–cACE complex structure was refined at 1.85 Å resolution with good reliability index values (R-factors) and geometry (Table 1).

**Table 1:** X-ray Data Collection and Refinement Statistics^a^

crystallographic statistics	Cip-cACE
resolution (Å)	1.85
space group	*P*2_1_2_1_2_1_
cell dimensions	
*a, b, c* (Å)	60.07, 85.38, 135.60
α, β, γ (°)	90.00, 90.00, 90.00
molecules per asymmetric unit	1
completeness (%)	100.0 (100.0)
*R*_merge_	0.258 (3.372)
*R*_pim_	0.067 (0.712)
⟨*I*/σ*I*⟩	6.7 (1.0)
CC_1/2_	0.995 (0.630)
multiplicity	13.4 (13.8)
*R*_work_/*R*_free_	0.20/0.23
RMSD bonds (Å)	0.008
RMSD angles (°)	1.786
Ramachandran angles (%)	
favored	98.22
allowed	1.78
outliers	0.00
average B-factors (Å^2^)	
cACE	34.78
Cip1	53.32
Cip2	45.43
water	36.17
number of non-hydrogen atoms	
cACE	4700
Cip1	24
Cip2	24
water	332
PDB code	9QAM

a Outer shell statistics are shown in brackets.

As previously shown, the overall structure of cACE is ellipsoidal, composed of two lobes, referred to as subdomains 1 and 2. The first 100 N-terminal residues make up the lid region, which is thought to open and close in order to allow access of substrates through the catalytic cleft^14,27,28^ (Figure 2A).

**Figure 2:**
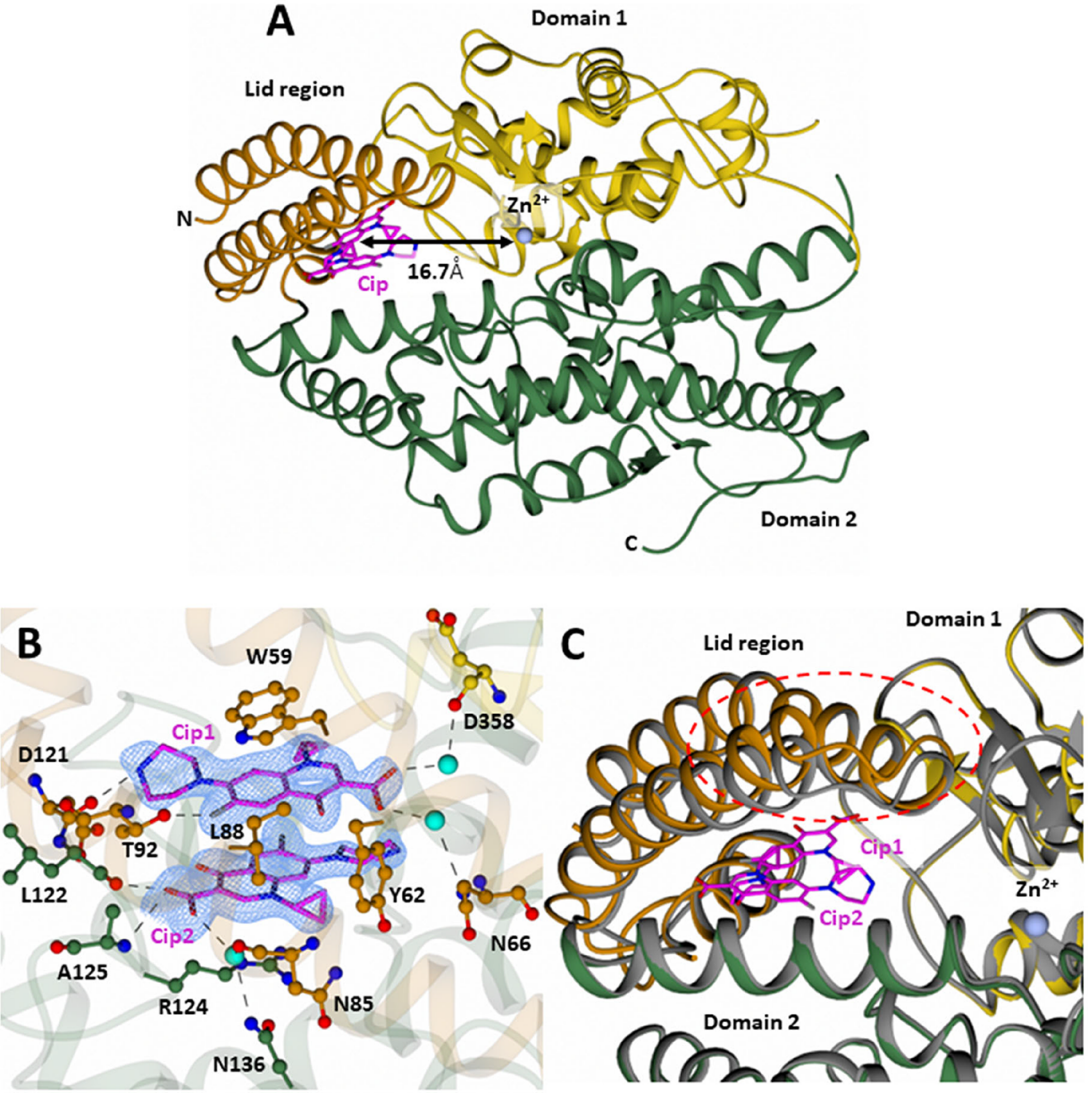
Structure of cACE in complex with Cip. (A) Cartoon representation of cACE in complex with Cip. The distance of the Cip binding site from the zinc binding catalytic pocket is shown by the black arrow. (B) Simulated annealing |Fo–Fc| omit electron density map for Cip (bound at the exosite of cACE) and Cip–cACE interacting residues. The map was contoured at 3 σ, colored in blue. Hydrogen bonds are shown by the dotted lines. (C) Comparison of the Cip-cACE structure to Apo-cACE (PDB code 2IUl). Cip-cACE is colored according to domains 1 and 2 (yellow and green, respectively), with the lid region shown in dark orange. The Apo-cACE structure is shown in gray, and Cip is in magenta.

At the core of the molecule is the active site composed of the HEXXH motif residues, His383, His387, and Glu411, that coordinate the catalytic zinc ion. Additionally, there are two chloride ions, Cl1 and Cl2, which coordinate residues Arg186, Trp485, Arg489, and Tyr224 and Arg522, respectively, away from the active site. In crystal structures of apo-cACE, there is nearly always a component from the purification or crystallization buffer bound to the active site. In the present structure, both a malonate ion (coordinating the zinc) and an N-carboxyalanine molecule (bound to the S1′ subsite) are bound to the active site. A large piece of positive difference map electron density (Figure 2B) was noted ∼16.7 Å opposite to the zinc binding site (Figure 2A) of the nonprimed region, which could readily be modeled as two antiparallel face-to-face π–π stacked Cip molecules. The two Cip molecules (Cip1 and Cip2) are sandwiched between Trp59 and Arg124 in a π–π–π–cation stacking interaction. Together, they interact with a total of 12 residues from the lid region, subdomain 1, and subdomain 2 (Table 2); however, most of the interacting residues are from the lid region of cACE (seven residues from the lid region).

**Table 2:**
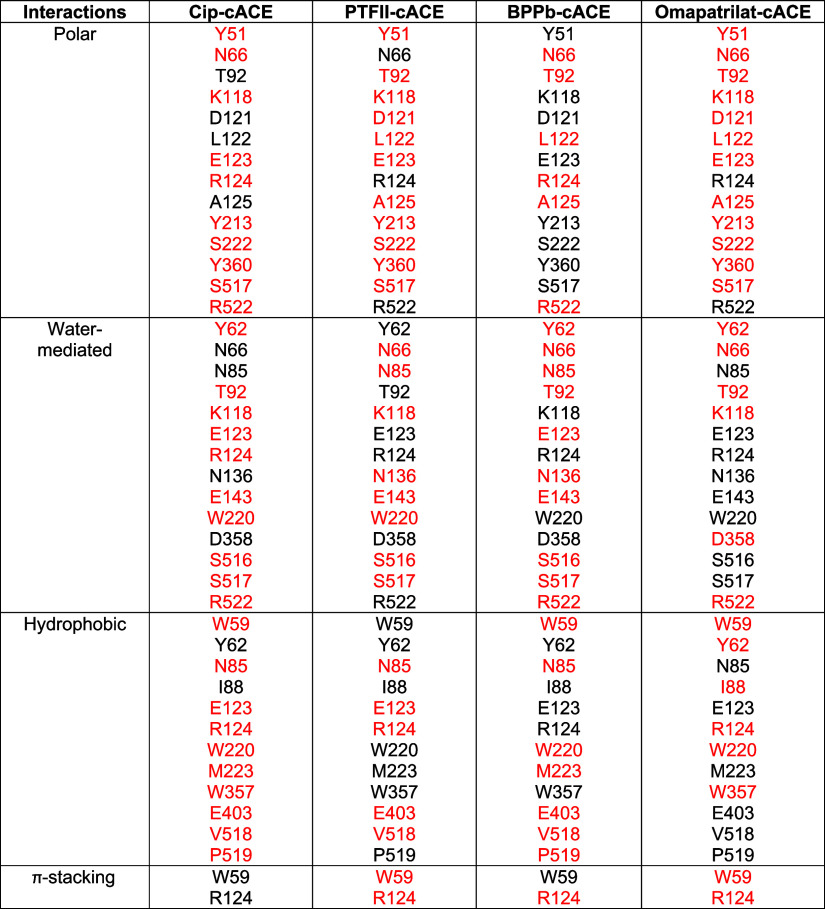
Comparison of cACE Exosite Amino Acid Residues Involved in Interactions with Cip, Phosphinic Tripeptide II-cACE, BPPb-cACE, and Omapatrilat^a^

a Interacting residues are shown in black, noninteracting ones are in red.

Cip1 forms additional interactions with Thr92 and Asp121, via hydrogen bonding, and Asp358 and Asn66 via a water-mediated interaction. Cip2 forms additional interactions with Leu122 and Ala125 via hydrogen bonding, Asn85 and Asn136 via a water-mediated interaction, and hydrophobic contacts with Ile88 and Tyr62 (Table 2 and Figure 2B). Superimposition of Cip-cACE with the apo-cACE structure (PDB 2IUL) results in an RMSD (for 558 Cα atoms) of 0.498 Å, indicating only minimal differences. This is likely due to the flexibility of the lid region, which appears to have subtly opened upon binding Cip (Figure 2C). The electron density of the lid region between residues 71 and 87, and higher B-factors, is indicative of a dual conformation between the closed and “partially open” state, likely influenced by the occupancy of the ligand; however, the quality of the electron density does not confer the accurate modeling of both conformations. The location of Cip binding between the lid region and subdomain 2 may therefore restrict the ability of the lid region to open and close fully in order for large substrates to bind.

A comparison of the Cip-cACE structure with nACE was performed to assess the feasibility of Cip binding to the equivalent location in nACE. Due to loss of Trp59 and Arg124, Cip binding at this location is not possible as the equivalent residues in nACE (nACE-Leu32 and nACE-Ser100, respectively) cannot form the π–π–π–cation stacking interaction observed in Cip-cACE. Superimposition of Cip-cACE with cACE in complex with phosphinic tripeptide F–II (PDB code 2XY9), bradykinin potentiating peptide B (PDB code 4APJ), and omapatrilat (PDB code 6H5W) reveals these inhibitors utilize similar residues distal to the active site (Table 2), except unlike Cip, they also bind the active site residues. Compared to Cip, the binding of phosphinic tripeptide F–II (PTFII) is driven by a series of interactions with a second PTFII zinc-anchored molecule, in what was termed a “handshake” motif. The space between Trp59 and Arg124 is smaller in the PTFII-cACE complex due to movement of both Trp59 and Arg124 toward PTFII and closure of the lid region (Figure 3A). PTFII forms a hydrophobic–hydrophobic-salt bridge stacking interaction instead (Figure 3B, Table 2). In addition, compared to Cip, it extends further toward both domain 1 by interacting with residue Trp357 and domain II by interacting with Met223, Pro519, and Trp220 ^29^ due to its greater length than Cip. This means that PTFII is occupying nearly the entire binding cavity, whereas Cip does not. The well-known bradykinin potentiating peptide b (BPPb) also occupies a large region of the catalytic pocket (Figure 3C) and forms a similar (but weaker) interaction compared to Cip (Table 2), where it is sandwiched between Trp59 and Arg124, consisting of a π–π-hydrophobic–hydrophobic interaction at this site (Figure 3D). Another potent ACEi, omapatrilat, was also shown to occupy this allosteric site (Figure 3E), where it forms an interaction with Arg124 but not Trp59 (Figure 3F). However, given the low occupancy, multiple conformations, and micromolar inhibition,^30^ its binding to this region is likely weak, and it does not make use of the strong interactions seen for Cip, PTFII, and BPPb (Table 2).

**Figure 3:**
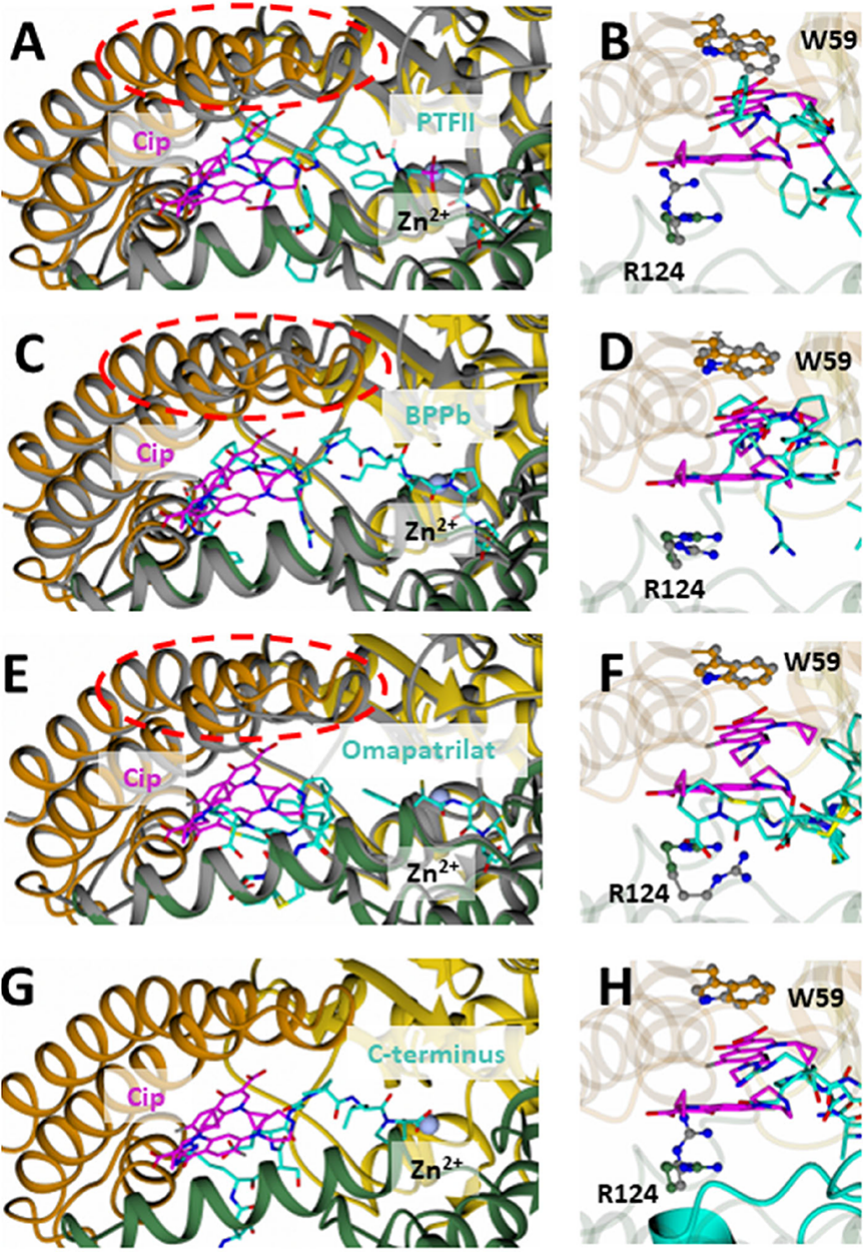
Details showing exosite binding of CiP with cACE in comparison with phosphinic peptide F–II (A,B), omapatrilat (C,D), BPPb (E,F), and the C-terminus inserted cACE structure (G,H). The difference in position of residues 71–87 of the lid region is highlight by the red circle. Cip is shown in magenta, and phosphinic peptide F–II, omapatrilat, BPPb, and C-terminus of the symmetry-related molecule are in cyan. Domains 1 and 2 are shown in yellow and green, respectively, with the lid region shown in dark orange. cACE of PTFII-cACE, BPPb-cACE, and Omapatrilat-cACE is shown in gray.

A recent native structure of cACE, in which the C-terminus of a symmetry-related molecule inserted into a hole within the lid region, demonstrated how cACE might be able to accommodate large substrates in a “closed state”. A comparison of the Cip-cACE structure and this structure indicates the binding of Cip would restrict access of substrates through this hole by steric hindrance (Figure 3G,H), providing insight into the potential mechanism of allosteric inhibition.

## Discussion

The design of allosteric inhibitors that target exosites distal to the active site of ACE is a promising strategy for modulating enzymatic activity, with potential therapeutic benefits. Conventional ACEi act by competitively binding to the zinc active site to directly inhibit enzymatic function. In contrast, allosteric inhibition offers an alternative mechanism by engaging structurally distinct regions of the enzyme, enabling functional modulation without directly obstructing substrate binding at the active site.

Allosteric regulation can either induce conformational changes that alter enzymatic activity or sterically inhibit large substrates distal to their scissile bond. In the case of ACE, exosites away from the zinc active site represent potential allosteric binding sites for modulating its activity. These allosteric inhibitors confer several advantages over conventional competitive inhibitors, including the potential for enhanced specificity, a lower likelihood of resistance emergence, and the capacity to modulate enzymatic activity with greater precision without inducing complete inhibition.

A potential target for allosteric inhibition of ACE is cACE, which is involved in substrate recognition and binding. This domain interacts with substrates such as Ang I and bradykinin, facilitating their cleavage by the active site zinc ion. Disruption of these interactions through allosteric inhibition could impair substrate binding and processing, thereby modulating ACE activity. In particular, these large substrates (Ang I, which is 10 amino acids long, and bradykinin, 9 amino acids long) would potentially recognize residues far from the active site in order to bind. Indeed, previous crystal structures, in particular, the crystal structures of cACE in complex with Ang II (1–8) and BPPb, have indirectly indicated this. Due to the catalytic turnover of Ang I (1–10) and bradykinin by cACE, a detailed mechanism of their binding has yet to be determined; however, cACE in complex with Ang II (1–8) has been reported. This structure revealed a “sliding” mechanism, in which the peptide occupied two conformations as it is “threaded” through a catalytic pocket.^31^ Furthermore, a comparison with the Cip-cACE structure revealed that the N-terminal end of Ang II is directed toward both the binding site of Cip and the nonprime hole within the lid region. However, the electron density was not sufficient to model beyond six residues of Ang II. The BPPb-bound cACE structure did show binding beyond this point, where it overlaps with the Cip binding site, indicating the plausibility that Ang I too would bind to this region, given the similarity at the N-terminus. An allosteric inhibitor binding to this region would therefore potentially restrict the correct positioning of the downstream C-terminal residues of Ang I for efficient cleavage at the active site or provide an additional barrier for its binding, thus reducing the rate of catalysis. This is evidenced by the Cip-cACE complex structure and kinetic results presented here, where the binding of Cip to the nonprime lobe clearly results in the selective inhibition of cACE (*K*_i_: 33.8 μM) without disrupting residues involved in binding close to the catalytic zinc ion.

The potential of repurposing Cip itself as a selective cACE inhibitor has many caveats to consider. First, Cip has been reported to cause adverse effects such as kidney failure^32,33^ and a heightened risk of aortic rupture,^34^ particularly when used in conjunction with the clinically administered ACEi enalapril^35^ and lisinopril.^36^ The data presented here suggest that such adverse events could be a result of binding at both the active site (enalapril/lisinopril) and the allosteric site (Cip) of cACE, given the moderate cACE selectivity of both enalapril and lisinopril. If indeed the adverse effects of Cip purely stem from its use alongside potent ACEi, which bind the active site, Cip has the potential to be repurposed as an ACEi if used alone to reduce side effects. However, this requires further investigation to rule out any other multidrug interactions that may be present. This is often difficult, as patients being treated with both Cip and ACEi are likely to display a range of comorbidities. Second, the impact of increased Cip use on antimicrobial resistance must be considered. Cip is an incredibly useful broad-spectrum antibiotic used to treat pneumonia, sexually transmitted infections, eye infections, and ear infections and as a preventative for meningitis. For that reason, its use (and other fluoroquinolones) has been recommended as a second-line therapy with the use of narrower-spectrum antibiotics prior. In that context, Cip may provide a good “template” for which better allosteric ACEi could be designed.

The crystal structure of cACE in complex with Cip, and the kinetic data presented here, along with the structural data of cACE in complex with phosphinic tripeptide II, BPPb, and omapatrilat, provides a starting point for the structure-based design of a cACE-specific allosteric inhibitor with the potential of reducing side effects.

## Conclusions

Traditional ACEi bind to the central catalytic zinc ion of individual domains, thereby inhibiting substrate turnover through competitive inhibition. In this study, we show that the widely used antibiotic, Cip, can selectively inhibit cACE activity by binding to an allosteric site ∼16.7 Å from the catalytic zinc ion. This structure serves as the basis for which new cACE selective allosteric ACEi can be designed.

Further extensive experimental study is required to understand the implications of repurposing Cip or its derivatives, where caution should be taken with respect to its use in conjunction with currently available ACEi and the impact that this would have on antimicrobial resistance.

## Data Availability

Atomic coordinates and structure factors have been deposited in the Protein Data Bank under accession code 9QAM for the Cip-cACE complex structure.

## Abbreviations

ACE: angiotensin I-converting enzyme
sACE: somatic angiotensin I-converting enzyme
tACE: testis angiotensin I-converting enzyme
nACE: angiotensin I-converting enzyme N-domain
cACE: angiotensin I-converting enzyme C-domain
Ang I: angiotensin I
Ang II: angiotensin II
Cip: ciprofloxacin
ACEi: ACE inhibitor
